# Mycobacterial Lineages Causing Pulmonary and Extrapulmonary Tuberculosis, Ethiopia

**DOI:** 10.3201/eid1903.120256

**Published:** 2013-03

**Authors:** Rebuma Firdessa, Stefan Berg, Elena Hailu, Esther Schelling, Balako Gumi, Girume Erenso, Endalamaw Gadisa, Teklu Kiros, Meseret Habtamu, Jemal Hussein, Jakob Zinsstag, Brian D. Robertson, Gobena Ameni, Amanda J. Lohan, Brendan Loftus, Iñaki Comas, Sebastien Gagneux, Rea Tschopp, Lawrence Yamuah, Glyn Hewinson, Stephen V. Gordon, Douglas B. Young, Abraham Aseffa

**Affiliations:** Author affiliations: Armauer Hansen Research Institute, Addis Ababa, Ethiopia (R. Firdessa, E. Hailu, B. Gumi, G. Erenso, E. Gadisa, T. Kiros, M. Habtamu, J. Hussein, R. Tschopp, L. Yamuah, A. Aseffa);; Animal Health and Veterinary Laboratories Agency, Weybridge, UK (S. Berg, G. Hewinson);; Swiss Tropical and Public Health Institute, Basel, Switzerland (E. Schelling, J. Zinsstag, S. Gagneux, R. Tschopp);; University of Basel, Basel (E. Schelling, J. Zinsstag, S. Gagneux); Imperial College, London, London, UK (B.D. Robertson, D.B. Young);; Addis Ababa University, Addis Ababa (G. Ameni);; University College Dublin Conway Institute, Dublin, Ireland (A.J. Lohan, B. Loftus, S.V. Gordon);; National Institute for Medical Research, London (I. Comas, D.B. Young);; Centre for Public Health Research, Valencia, Spain (I. Comas);; Centro de Investigación y Educación en Red en Epidemiología Biomédica Pública, Madrid, Spain (I. Comas)

**Keywords:** Tuberculosis and other mycobacteria, tuberculosis, Mycobacterium tuberculosis, Mycobacterium bovis, bacteria, bovine, pulmonary tuberculosis, extrapulmonary tuberculosis, TB lymphadenitis, cervical lymph nodes, lymph nodes, lineage, zoonoses, zoonotic transmission, Ethiopia

## Abstract

Molecular typing of 964 specimens from patients in Ethiopia with lymph node or pulmonary tuberculosis showed a similar distribution of *Mycobacterium tuberculosis* strains between the 2 disease manifestations and a minimal role for *M. bovis*. We report a novel phylogenetic lineage of *M. tuberculosis* strongly associated with the Horn of Africa.

Ethiopia is among the countries with the highest incidence of tuberculosis (TB) and has a yearly incidence of 261 cases/100,000 population. TB lymphadenitis in cervical lymph nodes (TBLN) accounts for ≈33% of all new cases in this country, which is greater than the global average of ≈15% (*1*). Ethiopia has the largest livestock population in Africa (≈51 million cattle), and recent studies have shown that bovine TB is endemic in this country (estimated prevalence 1%–10%) (*2*).

To explore the public health risk for bovine TB in Ethiopia, we have used molecular typing to characterize mycobacterial isolates from persons with TBLN and pulmonary TB who were visiting hospitals throughout the country. Our aim was to define the role of *Mycobacterium bovis* in human TB and to define the overall structure of the *M. tuberculosis* complex in Ethiopia.

## The Study

Patients with suspected TBLN or pulmonary TB who came to hospitals or health centers in study sites and provided voluntary consent were recruited into the study during 2006–2010. Fine needle–aspirate samples and sputum samples were collected from 2,151 patients attending hospitals in Gondar, Woldiya, Ghimbi, Butajira, and Negelle, Ethiopia. In addition, sputum samples were collected from patients at hospitals in Fiche, Jinka, and Filtu and at health centers at 3 suburban sites in Addis Ababa (Holeta, Sululta, and Chancho). Samples were cultured on Löwenstein-Jensen medium supplemented with glycerol or pyruvate and on modified Middlebrook 7H11 medium optimized for culture of *M. bovis*.

We characterized isolates belonging to the *M. tuberculosis* complex by using multiplex PCR for large sequence polymorphisms (*3*,*4*), spoligotyping (*5*), and lineage-specific single-nucleotide polymorphism analysis (*4*,*6*). Isolates of selected spoligotypes were characterized by 24-loci mycobacterial interspersed repetitive unit–variable number tandem repeat (MIRU-VNTR) analysis (*7*). Four *M. tuberculosis* isolates from a group of 36 with unusual spoligotype patterns were further characterized by genome sequencing (Illumina Inc., San Diego, CA, USA). Sequencing reads were mapped to the inferred most recent common ancestor of the *M. tuberculosis* complex (*6*). A final alignment of 13,199 single-nucleotide polymorphism positions was generated and analyzed by using the neighbor-joining method with a Tamura-Nei evolutionary model (www.megasoftware.net/mega_papers.php). Nontuberculous mycobacteria were characterized by sequencing of the 16S rDNA gene.

Characteristics of 964 cultures positive for acid-fast bacilli are summarized in Table 1. Most of these isolates had an intact RD9 region, which identified them as *M. tuberculosis*. Only 4 (0.4%) of 964 isolates had undergone RD9 and RD4 deletions characteristic of *M. bovis* (*3*). The 4 *M. bovis* isolates were obtained from cases of pulmonary TB, 3 of which were from patients living in pastoralist communities in southern Ethiopia. The 10 nontuberculous mycobacterial isolates were identified as *M. intracellulare*, *M. flavescens*, and *M. simiae*; 2 of the isolates were from patients co-infected with *M. tuberculosis*.

**Table 1:** *Mycobacterium spp.* and strains, Ethiopia, 2006–2010*

Collection site	Pulmonary TB							TBLN						
	*M. tuberculosis*‡					*M. bovis*	NTM	*M. tuberculosis*‡					*M. bovis*	NTM
	Total	L4	L3	L7	L1			Total	L4	L3	L7	L1		
Gondar	92	32	58	2	0	0	0	32	21	11	0	0	0	0
Woldiya	23	9	10	4	0	0	0	110	54	43	13	0	0	5
Ghimbi	47	40	6	1	0	0	0	69	58	11	0	0	0	0
Fiche‡	169	128	35	6	0	1	0	NA	NA	NA	NA	NA	NA	NA
Addis Ababa‡	60	50	7	3	0	0	0	NA	NA	NA	NA	NA	NA	NA
Butajira	70	60	9	1	0	0	0	110	91	13	6	0	0	0
Negelle/Filtu/Jinka	161	123	28	0	10	3	3	7	5	1	0	1	0	2
Total isolates	622	442	153	17	10	4	3	328	229	79	19	1	0	7
Total *M. tuberculosis* %	100	71.1	24.6	2.7	1.6	NA	NA	100	69.8	24.1	5.8	0.3	NA	NA

*Values are no. isolates. TB, tuberculosis; TBLN, TB lymphadenitis in cervical lymph nodes; NTM, nontuberculous mycobacteria; NA, not applicable. †Lineage of *M. tuberculosis* is expressed by an L followed by a digit. ‡TBLN samples were not collected in Fiche and Addis Ababa.

**Figure 1:**
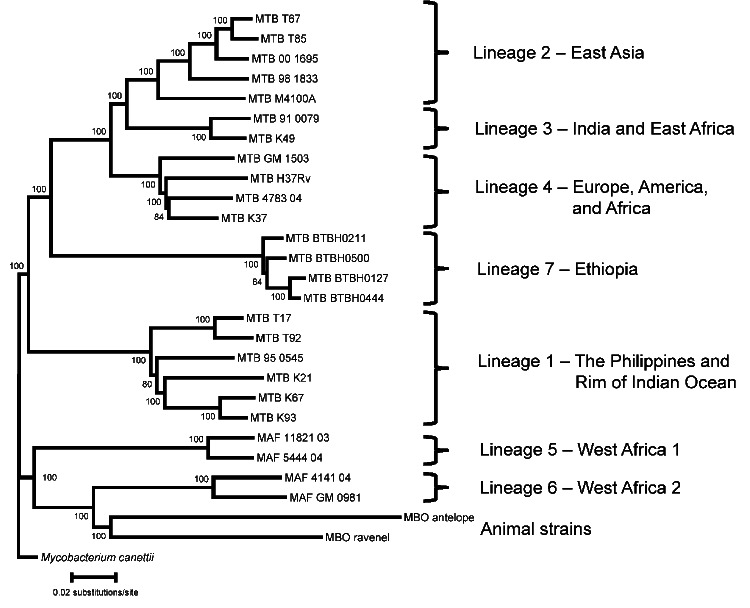
Lineages of the *Mycobacterium tuberculosis* (MTB) complex, Ethiopia, 2006–2010. Genome sequence analysis of 4 strains representative of 36 related isolates identified them as members of a new phylogenetic lineage (lineage 7) of *M. tuberculosis*, which has a phylogenetic location intermediate between ancient lineage 1 and modern Lineages 2, 3, and 4, and a branch point before the deletion of the TbD1 region (*3*). Nomenclatures for lineage names and numbers are as proposed (*4*,*6*). Phylogeny shown is based on 13,199 nt positions that were variable in at least 1 of the 28 *M. tuberculosis* complex strains represented in the tree. Numbers near nodes indicate percentage of bootstrap replicates supporting the topology after 1,000 pseudoreplicates. MAF, *M. africanum*; MBO, *M. bovis*.

Among the 954 isolates belonging to the *M. tuberculosis* complex, 671 (71%) belonged to lineage 4, which was the most common lineage in Ethiopia. However, lineage 3 was most prevalent in the northern sites of Gondar and Woldiya (122/257, 47%). Eleven strains belonging to lineage 1 were isolated in the southern region. Two isolates with a characteristic Beijing family spoligotype (spoligotype international type [SIT] 1) were identified as pseudo-Beijing strains belonging to lineage 3 (*8*). Thirty-six isolates with an unusual spoligotype pattern (missing spacers 4–24) and intact for the TbD1 region could not be assigned to known lineages. Genome sequencing identified these strains as members of a new lineage (lineage 7) localized between ancient lineage 1 and modern lineages 2, 3, and 4 of *M. tuberculosis* phylogeny (Figure 1). This new lineage 7 was prominent among strains collected in the Woldiya region (17/133 strains, 13%) (Table 1).

Lineage distribution was identical between the 2 disease forms at the national level; lineage 4 was isolated from 71% (442/662) and 70% (229/328) of pulmonary TB and TBLN patients, respectively, and lineage 3 was isolated from 25% (153/622) and 24% (79/328), respectively. The *M. tuberculosis* isolates encompassed 176 spoligotypes (Technical Appendix Table 1), of which 86 patterns were new to the international genotyping database 2 (SITVIT2) (www.pasteur-guadeloupe.fr:8081/SITVITDemo/) (*9*). A total of 11% (101/950) of the isolates represented single spoligotypes, and 62% (591/950) were included in 10 major spoligotype clusters (Table 2).

**Table 2:** Major spoligotype clusters of *Mycobacterium tuberculosis*, Ethiopia, 2006–2010*

SIT no.	SITVIT2	Lineage	No. isolates from each site							Disease	
			Go	Wo	Gi	Fi	AA	Bu	NFJ	PTB	TBLN
149	T3-ETH	4	9	17	3	48	15	20	38	125	25
25	CAS1	3	38	32	11	18	3	13	10	77	48
53	T1	4	7	10	17	10	8	32	14	58	40
37	T3	4	2	2	23	8	7	9	10	39	22
3134	New	4	1	1	3	4	2	33	0	26	18
26	CAS1	3	7	3	2	8	4	4	4	25	7
21	CAS1	3	5	2	1	3	0	0	7	16	2
41	LAM7-TUR	4	3	0	3	5	0	0	6	12	5
1729	Undesignated	7	0	13	0	3	0	1	0	5	12
4	LAM3	4	1	3	0	2	0	1	9	11	5
910	Undesignated	7	1	3	1	2	3	4	0	10	4

*SIT, spoligotype international type; SITVIT2, SIT international genotyping database 2; Go, Gondar; Wo, Woldiya; Gi, Ghimbi; Fi, Fiche; AA, Addis Ababa; Bu, Butajira; NFJ, Negelle, Filtu, and Jinka; PTB, pulmonary TB; TBLN, TB lymphadenitis in cervical lymph nodes.

**Figure 2:**
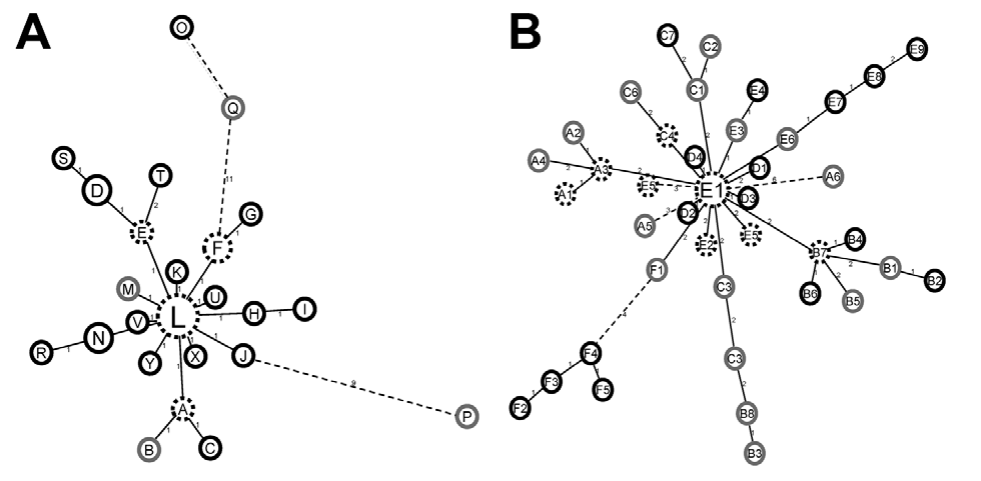
Mycobacterial interspersed repetitive unit–variable number tandem repeat (MIRU-VNTR) networks of major spoligotype clusters, Ethiopia, 2006–2010. Two large spoligotype clusters from lineage 4 (A) (90 isolates of sopligotype 149) and lineage 3 (B) (73 isolates of spoligotype 25) were further characterized by 24-loci MIRU-VNTR typing (Technical Appendix Table 2). Minimum-spanning trees were calculated for each cluster by using MIRU-VNTR*plus* (www.miru-vntrplus.org). Each circle indicates an individual genotype. Genotypes L and E1 include >15 isolates and remaining genotypes include <15 isolates. Genotypes indicated by a black circle were isolated from patients with pulmonary tuberculosis (TB); those in light gray were isolated from patients with TB lymphadenitis in cervical lymph nodes (TBLN); and those in dashed circles were isolated from patients with pulmonary TB and those with TBLN. Numbers on lines between circles indicate distance between 2 genotypes.

There was no difference in cluster distribution between pulmonary TB and lymph node TB isolates; 10% and 11%, respectively, were single types, and 64% and 60%, respectively, were included in dominant clusters. Two large clusters representative of lineage 4 (SIT 149) and lineage 3 (SIT 25) were further characterized by MIRU-VNTR typing (Technical Appendix Table 2) and network analysis (Figure 2). In each case, TBLN and pulmonary TB samples were dispersed throughout the network of spoligotype clusters.

All 4 *M. bovis* isolates from humans showed typical bovine spoligotype profiles lacking spacers 3, 9, 16, and 39–43. In addition, they lacked spacers 4–7 and had deletions of RDAf2, which are features that define strains of the African 2 clonal complex of *M. bovis* reported from TB-infected cattle in Ethiopia (*10*).

## Conclusions

The frequency of *M. bovis* in persons in this study (0.4%) is similar to that found in other studies of human TB in Africa (*11*) and South and Central America (*12*), but much lower than that observed among selected populations in Tanzania (16%) (*13*), Ethiopia (17%) (*14*), and Mexico (28%) (*15*). These findings indicate that the overall contribution of *M. bovis* to human TB is minor but greater in specific areas. In Ethiopia, monitoring of zoonotic transmission is needed in urban areas with high rates of bovine TB associated with intensive farming of imported dairy cattle (R. Firdessa et al., unpub. data) and among pastoralist populations from which human *M. bovis* cases were identified in this study.

Zoonotic transmission of *M. bovis* can be excluded as the predominant cause of the high national incidence of TBLN in Ethiopia. Mapping of disease networks by spoligotyping and MIRU-VNTR analysis showed an integrated distribution of the 2 disease forms, which suggested that cases of TBLN arise from within the pulmonary TB transmission network, rather than from an external zoonotic source.

We identified a novel phylogenetic lineage of M. tuberculosis (lineage 7) in multiple sites and at a high frequency in Woldiya in the northeastern highlands of Ethiopia. Screening of the SITVIT2 database (*9*) and the US Centers for Disease Control and Prevention National Tuberculosis Genotyping Surveillance Network Database (L.S. Cowan, pers. comm.) identified 23 (0.03%) of ≈90,000 isolates as members of lineage 7; all were isolated from patients whose country of origin (when known) was in the Horn of Africa. Lineage 7 is of considerable evolutionary interest because it represents a phylogenetic branch intermediate between the ancient and modern lineages of *M. tuberculosis* (*3**,**4**,**6*).

Technical Appendix Strain frequencies of respective spoligotype international types (SITs) of *Mycobacterium tuberculosis* isolated in this study, results of large and small sequence polymorphism typing, and typing results of 24-loci mycobacterial interspersed repetitive unit–variable number tandem repeats for isolates of spoligotypes SIT 25 and SIT 149, Ethiopia, 2006–2010.
